# The gland that never descended: Preoperative transarterial embolization of a symptomatic lingual thyroid

**DOI:** 10.1016/j.radcr.2026.09.014

**Published:** 2026-09-28

**Authors:** Orlando Díaz, Helio García

**Affiliations:** Department of Neuroradiology Neurointerventional Surgery, Houston Methodist, Houston, TX, USA

**Keywords:** Lingual thyroid, Ectopic thyroid, Transarterial embolization, Lingual artery, Base of tongue, Interventional radiology

## Abstract

Ectopic lingual thyroid is a rare vascular developmental anomaly that frequently represents the patient's sole functioning thyroid tissue. We report a 43-year-old euthyroid woman (baseline TSH 1.32 µIU/mL, free T4 1.12 ng/dL) with a symptomatic lingual thyroid measuring 2.1 × 1.6 × 2.4 cm on CT presenting with progressive dysphagia, hoarseness, and globus sensation. Because the hypervascular tongue-base mass required resection, preoperative transarterial embolization of its bilateral lingual arterial supply (right-dominant) was performed via a right transfemoral approach using a Scepter C 4 × 10 balloon microcatheter with 1 vial of 300–500 µm polyvinyl alcohol (PVA) particles followed by Onyx 18 (1 cc per pedicle, total 3 cc; 0.5 cm reflux tolerated), achieving near-complete devascularization without complications (fluoroscopy time: 40.7 minutes; cumulative reference air kerma: 1160 mGy). Single-port transoral robotic-assisted resection followed the next day; intraoperative direct laryngoscopy demonstrated marked mucosal blanching, and excision was completed with an estimated blood loss of only 5 mL without transfusions. Total resection of the ectopic tissue produced expected postsurgical hypothyroidism (TSH 11.0 µIU/mL, free T4 0.61 ng/dL), successfully managed with levothyroxine 100 µg daily; at two-month follow-up she remained euthyroid on a stable dose, with improved swallowing and resolution of globus. Transarterial embolization served as an effective preoperative devascularization adjunct that reduced intraoperative hemorrhage during transoral robotic resection of a hypervascular tongue-base lesion.

## Graphical abstract

**Figure:**
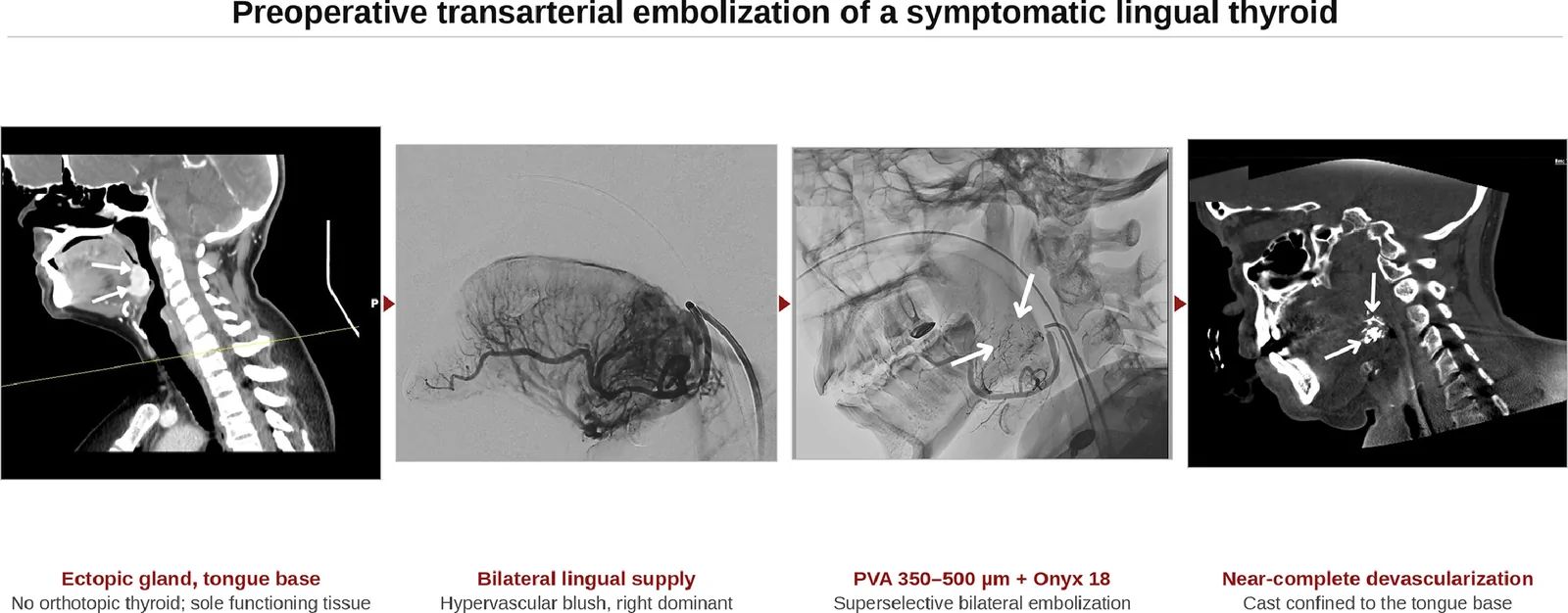

## Introduction

The thyroid is the first endocrine organ to form, budding in the fourth gestational week from the floor of the primitive pharynx and descending from the foramen cecum to its pretracheal position by the seventh week, the thyroglossal duct regressing in its wake (Fig.1) [1,2]. When descent fails, functioning tissue persists at the tongue base as a lingual thyroid — the most common thyroid ectopia (≈ 90%), rare in absolute terms (1:100,000–1:300,000), female-predominant, and, in roughly 70% of patients, the only functioning thyroid tissue (Table 1) [1,[3], [4], [5], [6]].

**Fig. 1:**
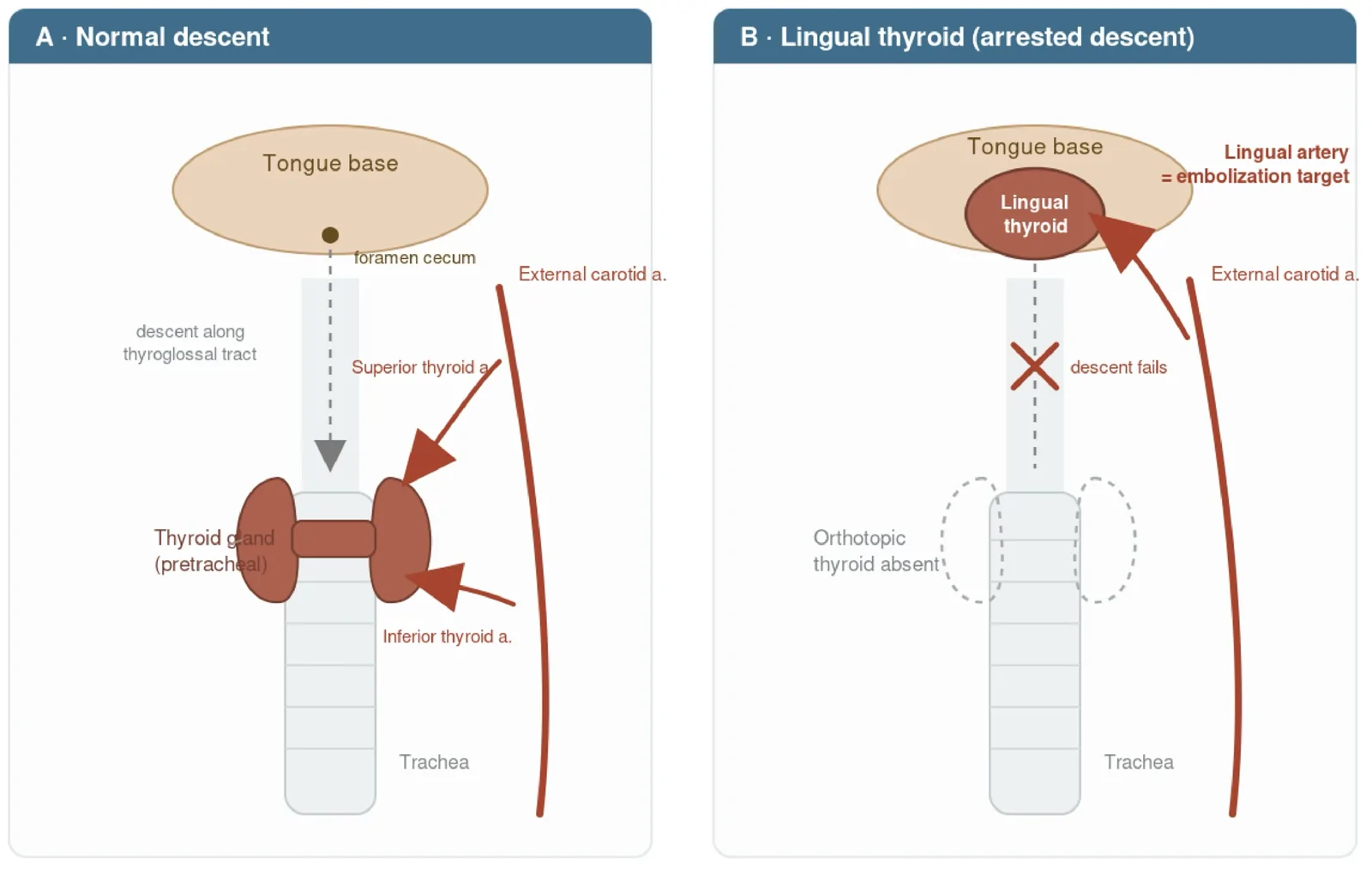
Thyroid descent and the origin of a lingual thyroid (schematic; not to scale). (A) Normal migration from the foramen cecum along the thyroglossal tract to the pretracheal position, where the gland is supplied by the superior and inferior thyroid arteries [1,2]. (B) Arrested descent leaves a lingual thyroid at the tongue base, retaining the primitive supply of the lingual artery, a branch of the external carotid — the anatomical basis, and target, for transarterial embolization [1].

**Table 1:** Epidemiology, physiology, vascular supply, and management of the lingual thyroid. TSH, thyroid-stimulating hormone.

Parameter	Value
**Clinical prevalence**	1:100,000 – 1:300,000 [1]
**Share of all ectopic thyroid**	≈ 90% [1,4]
**Female-to-male ratio**	≈ 4:1 (up to 7:1) [1]
**Sole functioning thyroid tissue**	≈ 70% of patients [1,6]
**Enlargement triggers**	Puberty, pregnancy, menopause (rising TSH) [1,4]
**Common symptoms**	Dysphagia, dysphonia, globus, dyspnea, bleeding [4,7]
**Dominant blood supply**	Lingual artery (external carotid branch); aberrant feeders possible [13,16]
**Management options**	Observation; levothyroxine suppression; radioiodine; surgery [1,10,11]; transarterial embolization [13,15]

Under the hormonal drive of puberty, pregnancy, or menopause the gland may enlarge and cause dysphagia, dysphonia, globus, and dyspnea, as well as severe hemorrhage [4,[7], [8], [9]]. Conventional treatments — levothyroxine suppression, radioiodine, and surgical or transoral robotic excision — each confront a densely vascular tongue-base field and the risk of rendering a sole-source patient athyreotic [3,[10], [11], [12]].

Because the gland is supplied chiefly by the lingual artery, it is accessible from the external carotid, and transarterial embolization has been used both for hemostasis and as a preoperative adjunct in the bleeding or hypervascular lingual thyroid [[13], [14], [15]], and more broadly to control tongue-base hemorrhage of other causes [16,17]. Its role — as definitive therapy, preoperative adjunct, or tissue-sparing alternative — remains poorly defined. We report a lingual thyroid managed with transarterial embolization, highlighting objective procedural metrics, radiation exposure, and quantitative surgical outcomes.

## Case presentation

### Patient information

A 43-year-old woman with a history of anxiety, depression, Barrett's esophagus, and prior Roux-en-Y gastric bypass. She had no previous head and neck surgery or radiation and no known thyroid disease; her maternal grandmother and great-grandmother had reportedly undergone thyroidectomy for unknown reasons.

### Presentation

She reported approximately one year of progressive dysphagia — particularly to larger pills and multivitamins — superimposed on several months of persistent throat pain, hoarseness, globus, and frequent throat clearing, together with constitutional complaints of insomnia and heat/cold intolerance. Her occupation required frequent speaking, which aggravated the symptoms.

### Timeline

Progressive dysphagia began approximately one year before presentation, superimposed on several months of throat pain, hoarseness, and globus. CT, thyroid scintigraphy, and flexible laryngoscopy established the diagnosis. Transarterial embolization was performed the day before surgery and transoral robotic resection the following day. Recovery was uneventful and the patient was discharged; postsurgical hypothyroidism was subsequently managed with levothyroxine, and improved swallowing was documented at two-month outpatient follow-up.

### Diagnostic work-up

CT of the neck demonstrated an enhancing midline mass at the base of the tongue measuring 2.1 × 1.6 cm in axial dimensions and 2.4 cm craniocaudally, exerting mass effect on the oropharyngeal airway, with absence of an orthotopic thyroid gland in the infrahyoid pretracheal space (Fig. 2). Thyroid scintigraphy with technetium and radioiodine demonstrated intense focal uptake at the tongue base with no activity in the normal pretracheal thyroid bed; the 4-hour iodine uptake was 6% (normal, 5–10%) and the 24-hour radioiodine uptake was 19.7% (normal, 10–25%). Flexible laryngoscopy demonstrated a rounded submucosal mass at the base of the tongue (Fig. 3). Baseline serum thyroid function testing confirmed a euthyroid state, with a TSH level of 1.32 µIU/mL (normal range, 0.45–4.5 µIU/mL) and a free T4 level of 1.12 ng/dL (normal range, 0.8–1.8 ng/dL). These findings established that the lingual thyroid was the patient's only functioning thyroid tissue.

**Fig. 2:**
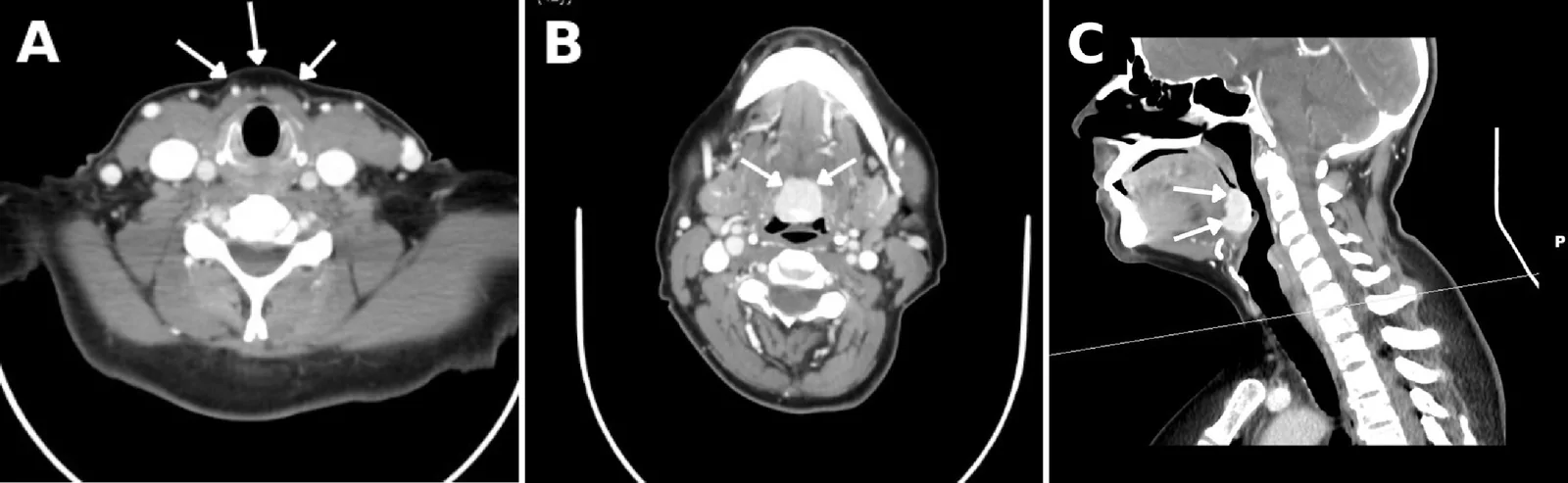
Contrast-enhanced CT of the neck. (A) Axial image at the infrahyoid level demonstrates complete absence of an orthotopic thyroid gland in the normal pretracheal space. (B) Axial and (C) sagittal reconstructed images demonstrate a well-defined, hyperattenuating midline mass at the base of the tongue measuring 2.1 × 1.6 cm in axial dimensions and 2.4 cm craniocaudally (arrows), causing mild mass effect and narrowing of the oropharyngeal airway, consistent with an ectopic lingual thyroid.

**Fig. 3:**
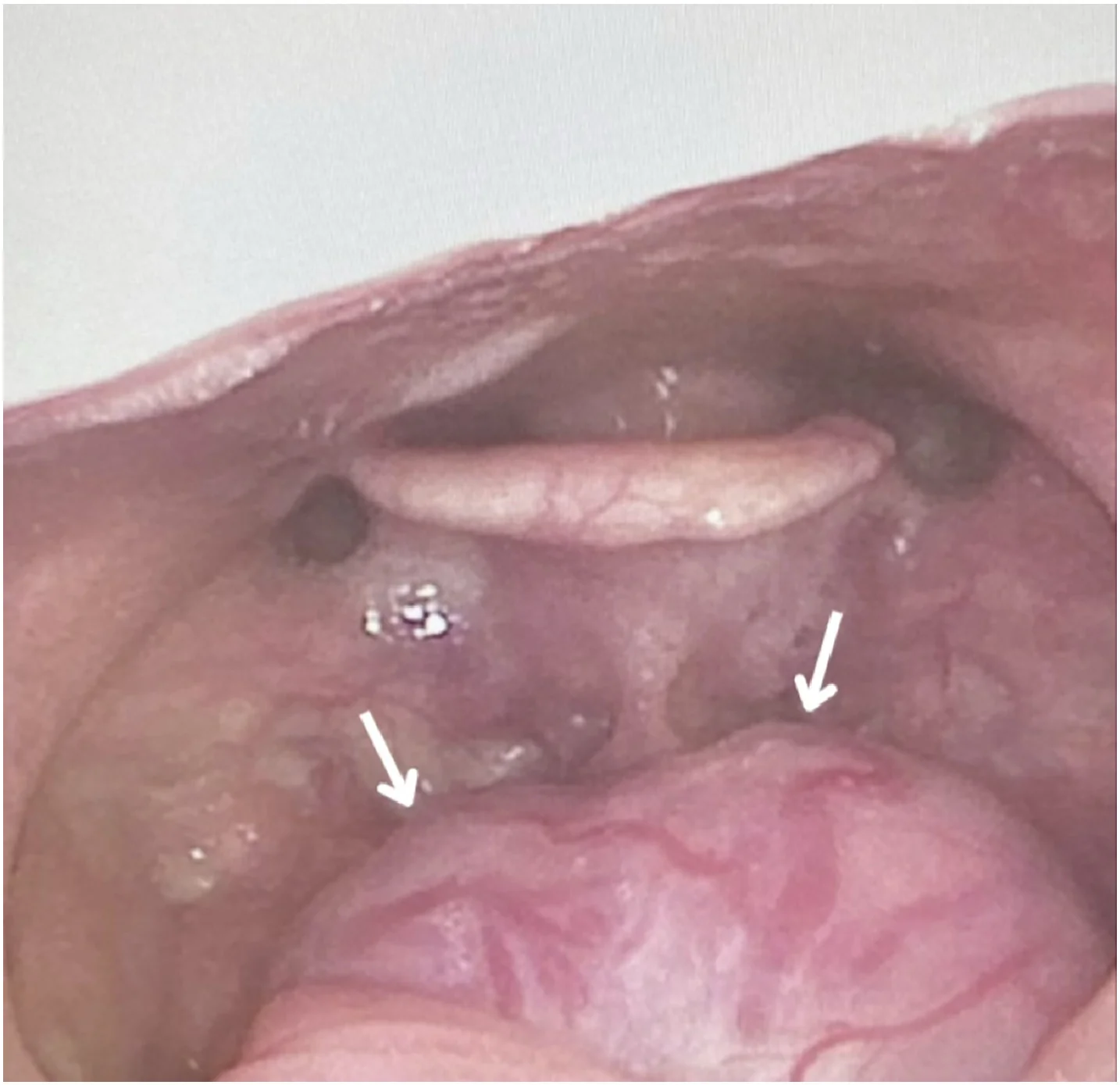
Flexible fiberoptic laryngoscopy. Rounded submucosal mass demonstrated at the base of the tongue protruding into the oropharyngeal airway.

### Indication and procedure

Because the hypervascular tongue-base gland required resection and posed a bleeding risk, preoperative transarterial embolization was performed the day before surgery to reduce intraoperative hemorrhage. A complete diagnostic cerebral arteriogram evaluating the bilateral internal carotid, external carotid, and vertebral arteries was performed prior to embolization to explicitly rule out dangerous anastomoses; no dangerous anastomoses were present in the lingual artery territory. Both lingual arteries (white arrow) were hypertrophied and supplied arterial feeders (white arrowhead) to a hypervascular ectopic gland (black arrow) at the tongue base, with the right lingual artery as the dominant supply (Fig. 4).

**Fig. 4:**
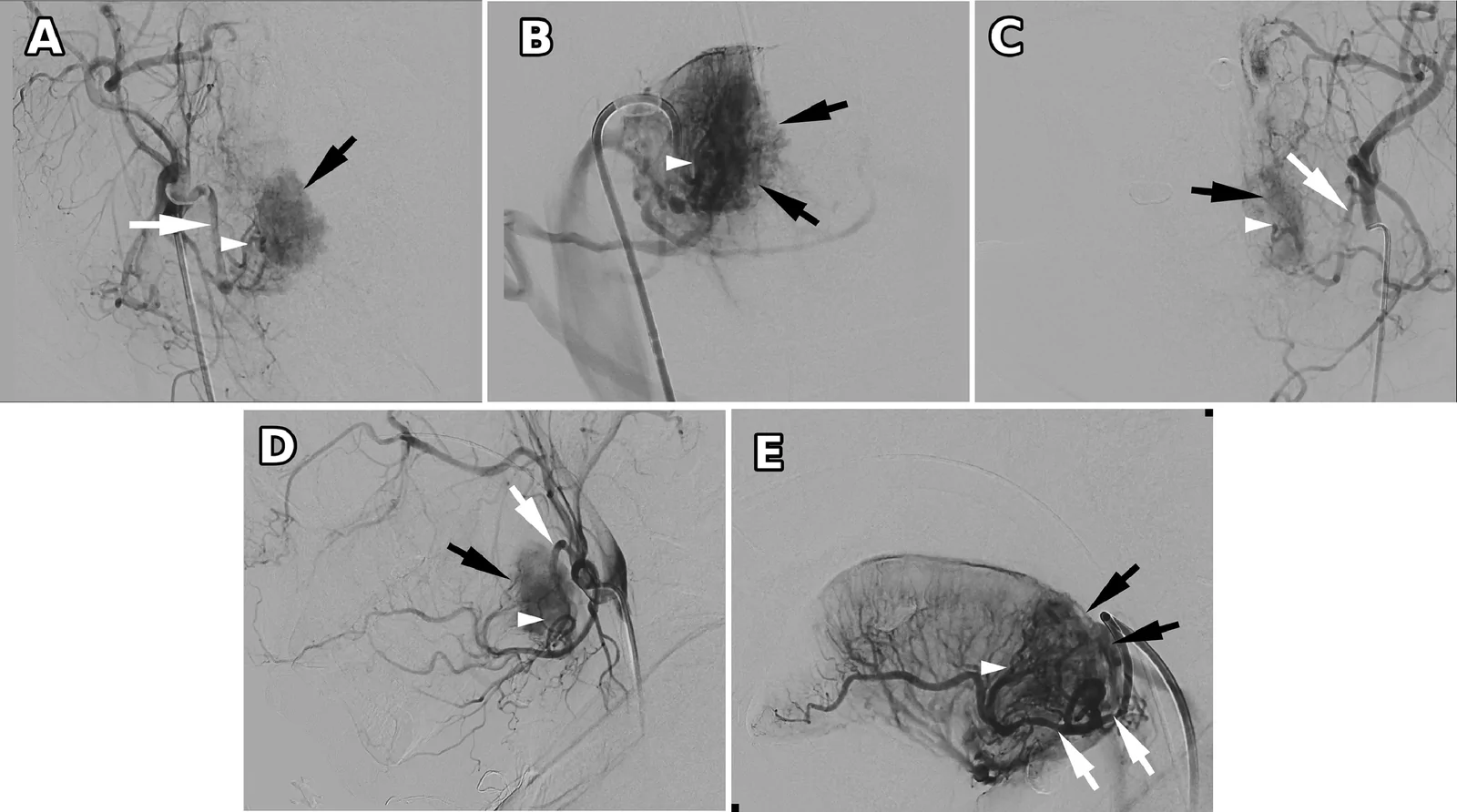
Diagnostic angiography. (A) Right external carotid artery injection, AP projection. (B) Right lingual artery injection, AP projection. (C) Left external carotid artery injection, AP projection, and (D) lateral projection. (E) Left lingual artery injection, lateral projection. All injections demonstrate bilateral supply to the ectopic gland, with dominant supply arising from the right lingual artery (B, E). Annotations identify the main lingual artery (white arrow), tumor feeding vessels (white arrowhead), and the hypervascular ectopic thyroid gland (black arrow).

From a right transfemoral approach, a Scepter C 4 × 10 balloon microcatheter was navigated super selectively into the bilateral lingual feeding branches (right dominant first, followed by left). To achieve deep microvascular bed penetration and parenchymal ischemia, 1 vial of 300–500 µm polyvinyl alcohol (PVA) microparticles was delivered first into the distal circulation. Subsequently, Onyx 18 liquid embolic agent was administered (1 cc per pedicle, total 3 cc) to accomplish solid proximal vessel occlusion and cast formation, with up to 0.5 cm of reflux tolerated (Fig. 5). Post-embolization external carotid angiography confirmed near-complete devascularization — defined as near-total resolution of the tumoral blush on selective external carotid injection — without contrast extravasation or non-target reflux, and DynaCT confirmed that the radiopaque Onyx cast was strictly localized throughout the entire ectopic gland at the tongue base (Fig. 6). Total fluoroscopy time was 40.7 minutes, with a cumulative reference air kerma of 1160 mGy. Hemostasis at the right femoral access site was achieved with an Angio-Seal closure device. There were no complications, and the neurological examination was unchanged.

**Fig. 5:**
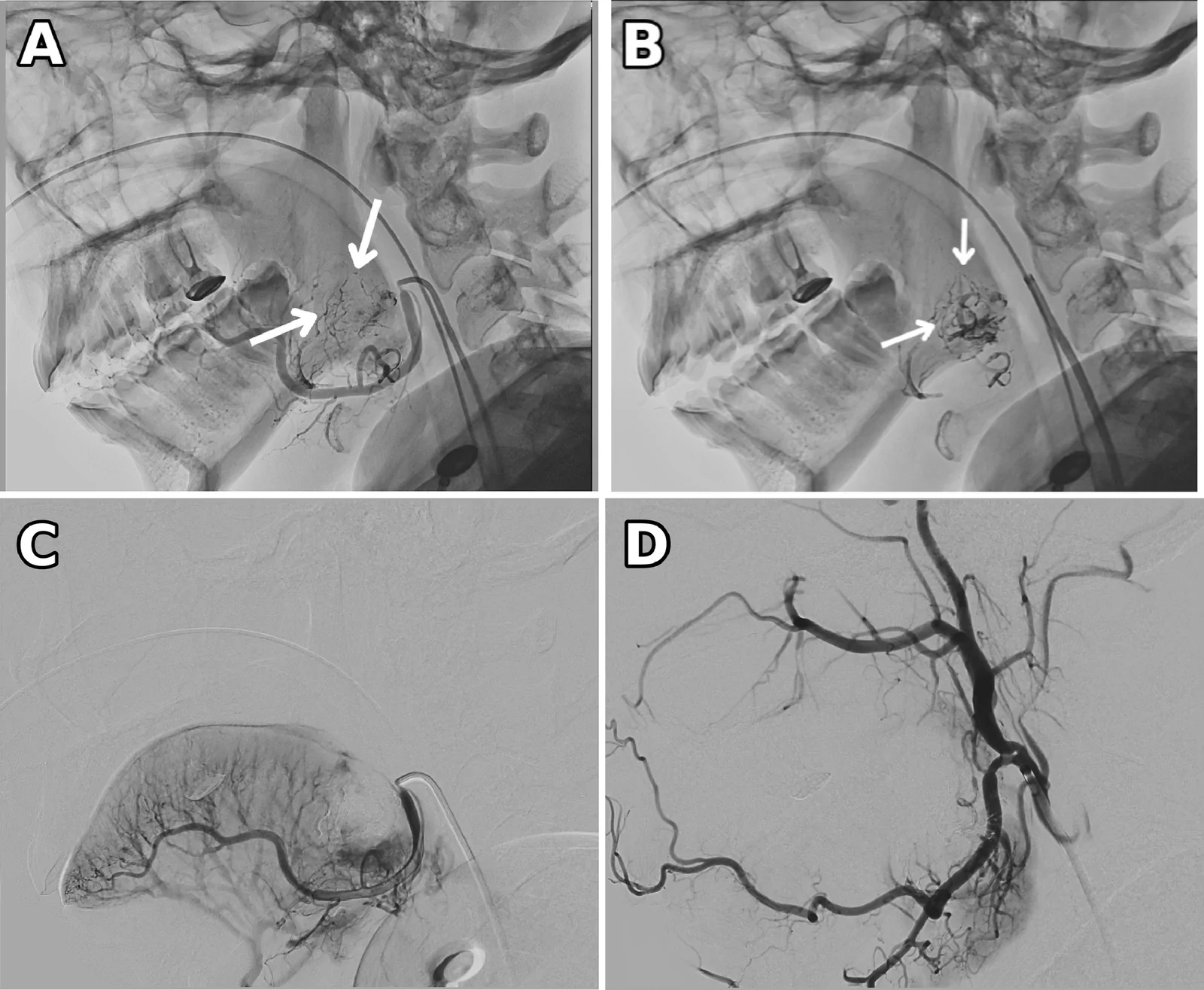
Post-embolization angiography. (A, B) Post-embolization unsubtracted fluoroscopic images demonstrating the radiopaque Onyx 18 cast occupying the vascular bed of the ectopic gland. (C, D) Post-embolization right and left selective external carotid arteriograms demonstrating near-complete devascularization with total resolution of the hypervascular tumoral blush without contrast extravasation or non-target reflux.

**Fig. 6:**
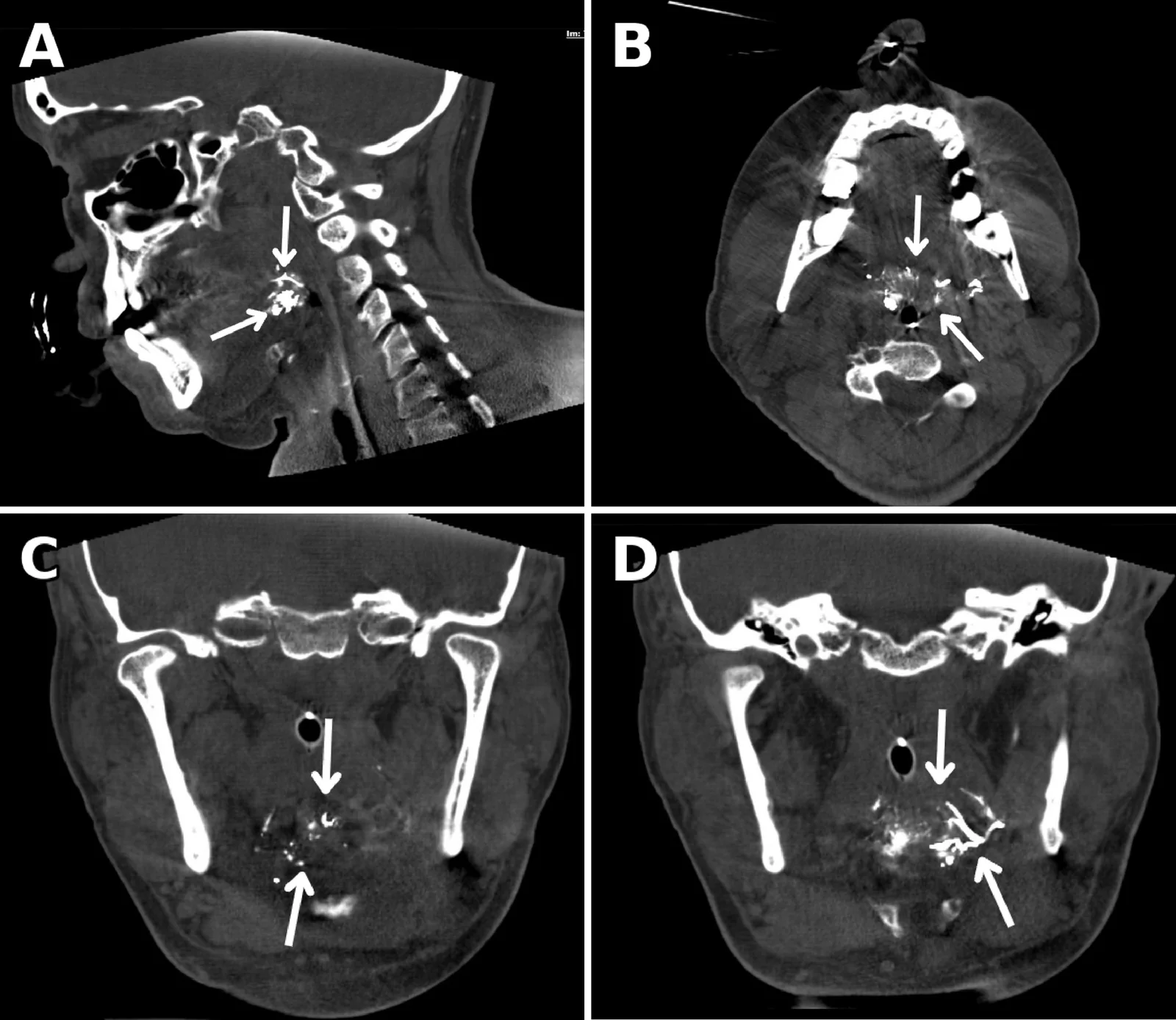
Post-embolization DynaCT of the neck. Reconstructed (A) sagittal, (B) axial, and (C, D) coronal images demonstrating the high-attenuation radiopaque Onyx 18 cast strictly localized within and corresponding to the entire embolized lingual thyroid gland at the base of the tongue, confirming complete glandular coverage and absence of non-target embolic reflux.

### Outcome and follow-up

The following day the patient underwent single-port transoral robotic (da Vinci SP) resection of the lingual thyroid, together with direct laryngoscopy, nasal endoscopy, esophagoscopy, and Dobhoff tube placement. Direct laryngoscopy before resection revealed marked mucosal blanching and ischemic devascularization of the ectopic gland resulting from the previous day's embolization. Setup and resection required an additional 60 minutes because of single-port maneuvering. The devascularization rendered the surgical field dry, with an estimated blood loss of 5 mL and no intraoperative transfusion. Recovery was uneventful, with return to baseline oral intake, and she was discharged in good condition. Because the excised ectopic gland was her only functioning thyroid tissue, she developed acquired primary postsurgical hypothyroidism (TSH 11.0 µIU/mL, free T4 0.61 ng/dL) and was started on levothyroxine 100 µg daily, with endocrinology follow-up. At two-month outpatient follow-up she reported improved swallowing and complete resolution of globus, and remained clinically and biochemically euthyroid on a stable levothyroxine dose of 100 µg daily, with no recurrent symptoms.

## Discussion

The lingual thyroid's embryology and vascular anatomy are inseparable: a gland that never descended retains the primitive supply of the tongue base, drawing on the lingual artery rather than the superior and inferior thyroid arteries [1,2]. This produces a superficial, hypervascular airway mass on an accessible external carotid pedicle — prone to severe bleeding and, for that reason, amenable to super selective embolization [13,16]. Angiography also resolves a diagnostic pitfall: a hypervascular tongue-base mass may be mistaken for malignancy or a vascular tumor [8,9], and blind biopsy or resection risks marked bleeding or iatrogenic athyreosis [13].

Conventional management must contend with brisk bleeding in a field that holds the patient's only functioning thyroid tissue [1,6,[10], [11], [12]]. Reported experience with embolization of the lingual thyroid itself (Table 2) spans hemostasis in the acutely bleeding gland, a preoperative adjunct before transoral robotic resection, and control of hemorrhage during pregnancy [[13], [14], [15]]; the same technique is well established for tongue-base hemorrhage of other causes [16,17]. In the present case, utilizing a Scepter C 4 × 10 balloon microcatheter to deliver 1 vial of 300–500 µm PVA microparticles first for distal capillary bed penetration followed by Onyx 18 (1 cc per pedicle, total 3 cc; 0.5 cm reflux) for proximal cast formation achieved near-complete devascularization, directly translating into an estimated intraoperative blood loss of 5 mL. Safety rested on a complete diagnostic cerebral arteriogram (evaluating bilateral internal carotid, external carotid, and vertebral arteries) to confirm the total absence of dangerous anastomoses in the lingual artery territory prior to embolization [13,16].

**Table 2:** Reported cases in which transarterial embolization was used in the management of a lingual thyroid, compared with the present case. The uniform absence of embolic-agent, angiographic-endpoint, and durability data (“not reported”) marks a gap that structured reporting should close. TORS, transoral robotic surgery.

Report (year)	Presentation	Treatment	Embolic material	Outcome	Learning point
**Chiu et al. (2002) [****15****]**	Bleeding lingual thyroid in pregnancy; sole functioning tissue	Bilateral lingual artery embolization, then excision via mandibular swing	Not reported	Hemostasis; delivered at 36 weeks, healthy neonate	Embolization enables safe excision when bleeding
**Filarski et al. (2021) [****13****]**	Life-threatening hematemesis; airway risk	Embolization of aberrant feeders + tracheostomy, then TORS	Not reported	Bleeding controlled; decannulated	Embolization plus minimally invasive resection
**Seo et al. (2022) [****14****]**	Progressive obstruction, refractory to levothyroxine	Preoperative right lingual artery embolization (3 days pre-op), then TORS	Not reported	Airway patent; no recurrence	Used as a preoperative adjunct; blood loss not reported
**Present case**	Symptomatic lingual thyroid; sole functioning thyroid tissue (dysphagia, hoarseness, globus)	Preoperative bilateral lingual artery embolization, then transoral robotic resection	PVA particles + Onyx 18	Near-complete devascularization; uneventful resection; postsurgical hypothyroidism on levothyroxine	Preoperative embolization as a bleeding-sparing adjunct, with explicit agent and endpoint

The distinction from ablation or resection is chiefly endocrine. Because the ectopic gland is usually the only functioning thyroid tissue, removing or destroying it commits the patient to lifelong replacement [1,6]. Devascularization without excision parallels thyroid artery embolization for benign goiter and Graves' disease, which achieves volume reduction while attempting to preserve hormone synthesis [[18], [19], [20]]. Whether embolization alone can reduce volume while preserving sufficient hormone synthesis in a lingual thyroid remains an unproven, investigational concept. In our patient surgical excision followed 24 hours after embolization, producing the expected postsurgical hypothyroidism (TSH 11.0 µIU/mL, free T4 0.61 ng/dL) managed with levothyroxine.

Fig. 7 consolidates these considerations into a proposed management pathway. It is a conceptual synthesis rather than a validated algorithm: the roles shown for embolization as definitive therapy or as a tissue-sparing alternative are explicitly designated investigational, distinct from its demonstrated role as a preoperative devascularization adjunct.

**Fig. 7:**
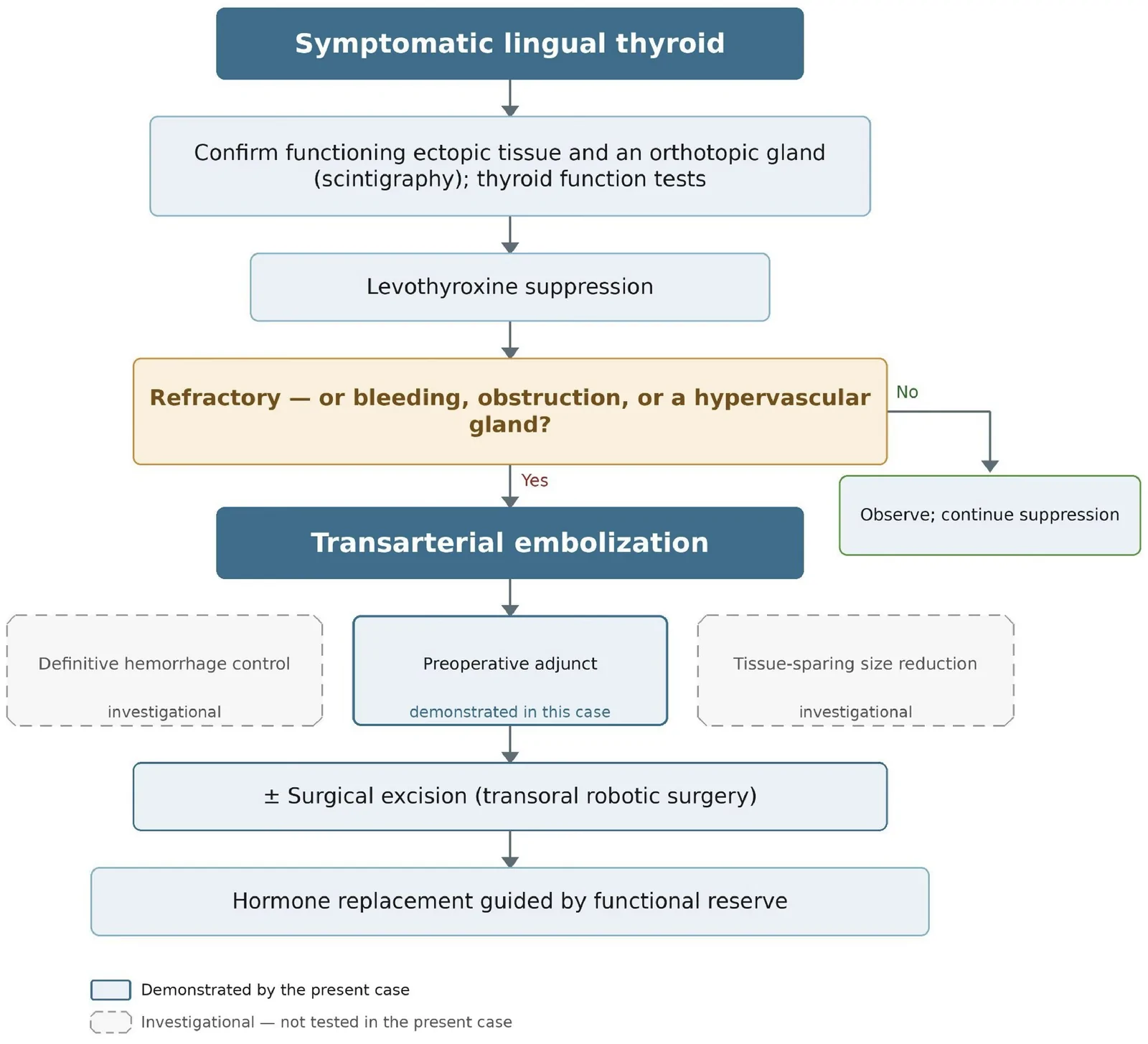
Proposed management pathway for the symptomatic lingual thyroid. Flowchart integrating scintigraphic assessment for a functioning orthotopic gland, levothyroxine suppression, and — for bleeding, obstruction, or a hypervascular gland — transarterial embolization as a preoperative adjunct. The pathways proposing embolization as definitive therapy or as a tissue-sparing alternative to resection are explicitly designated as investigational/hypothetical pathways, extrapolated from thyroid arterial embolization for benign goiter and Graves' disease.

These advantages are balanced by real hazards. External-to-internal carotid and vertebral anastomoses expose the patient to non-target embolization, stroke, and cranial-nerve injury, and aggressive tongue-base embolization may cause mucosal necrosis; super selective technique and mapping of dangerous anastomoses are mandatory [13,16]. Because the gland was resected the day after embolization, the present case cannot address whether devascularization alone preserves hormone output; that question requires patients managed with embolization alone and prospective functional follow-up. As a single case without long-term follow-up, our observations require confirmation, and the literature's silence on embolic agent, endpoint, and durability (Table 2) is a gap that structured reporting must close.

## Conclusions

Transarterial embolization appears to be a feasible preoperative devascularization adjunct in the management of symptomatic or hypervascular lingual thyroid, and in this case was followed by an estimated intraoperative blood loss of 5 mL during transoral robotic resection [[13], [14], [15]]. Explicit documentation of embolic agents, angiographic endpoints, radiation parameters, and quantitative surgical outcomes is essential to define standardized endovascular protocols for this rare entity.

## Ethical approval

Institutional review board approval was not required for this single-patient case report at our institution. The work was conducted in accordance with the Declaration of Helsinki.

## Data availability

All data supporting the findings of this case report are contained within the article. De-identified imaging is available from the corresponding author on reasonable request.

## Declaration of generative AI and AI-assisted technologies in the writing process

During the preparation of this work the authors used Claude (Anthropic) in order to assist with language editing, manuscript organization, and formatting. After using this tool, the authors reviewed and edited the content as needed and take full responsibility for the content of the publication. Artificial intelligence was not used for clinical decision-making, image interpretation, data analysis, or the generation of scientific conclusions.

## Prior presentation

None. This work has not been presented or published elsewhere.

## CRediT authorship contribution statement

**Orlando Díaz:** Conceptualization, Investigation, Methodology, Supervision, Writing – review & editing. **Helio García:** Conceptualization, Investigation, Data curation, Visualization, Writing – original draft, Writing – review & editing.

## Patient consent

Written informed consent was obtained from the patient for publication of this case report and any accompanying images.
